# The Costs of Digital Health Interventions to Improve Immunization Data in Low- and Middle-Income Countries: Multicountry Mixed Methods Study

**DOI:** 10.2196/62746

**Published:** 2025-08-18

**Authors:** Carlo Federici, Maria Verykiou, Marianna Cavazza, Willyhelmina Olomi, Piero Irakiza, Kizito Kayumba, Edith Rodriguez, Luis Enrique Castillo Mendoza, Stefano Malvolti, Claire Hugo, Nyanda Elias Ntinginya, Souleymane Camara, Issa Sabi, Nagnouma Sano, Hassan Sibomana, Jeanine Condo, Aleksandra Torbica, Claudio Jommi, Carsten Mantel, Viviana Mangiaterra

**Affiliations:** 1 Centre for Research on Health and Social Care Management (CERGAS), SDA Bocconi School of Management, Via Sarfatti 10, Milano, 20136, Italy; 2 NIMR Mbeya Medical Research Centre (NIMR-MMRC), Mbeya, Tanzania; 3 Center for Impact, Innovation and Capacity building for Health Information Systems and Nutrition (CIIC-HIN), Kigali, Rwanda; 4 Individual consultant, Tegucigalpa, Honduras; 5 MMGH Consulting, Zurich, Switzerland; 6 Ministère de la Santé de Guinée, Conakry, Guinea; 7 Rwanda Biomedical Center (RBC), Kigali, Rwanda; 8 Department of Pharmaceutical Sciences, Univerisità degli Studi del Piemonte Orientale, Novara, Italy

**Keywords:** electronic immunization registries, immunization, low- and middle-income countries, digital health intervention, digital health, data management, economic costs, implementation, survey

## Abstract

**Background:**

Digital health interventions, such as electronic immunization registries (eIRs) and electronic logistic management information systems (eLMIS), have the potential to significantly improve immunization data management and vaccine logistics in low- and middle-income countries (LMICs). Despite their growing adoption, there is limited evidence of the financial and economic costs associated with their implementation compared to traditional paper-based systems.

**Objectives:**

We aimed to measure the costs of implementing eIR and eLMIS systems in LMICs and to estimate their economic costs as compared to the previous paper-based registries.

**Methods:**

The study was conducted across four countries—Guinea, Honduras, Rwanda, and Tanzania—which implemented the tools in 2018, 2012, 2019, and 2014, respectively. A combination of primary and secondary data sources was used for the analysis. Retrospective cost data regarding the design, development, and implementation of the tools were directly obtained from implementers and National Immunization Program offices in all countries. Primary survey data were collected to gauge the operational expenses of immunization information systems, both with and without electronic tools, using an activity-based costing approach in 275 facilities. The annual cost of the immunization information system at the national level was then extrapolated and compared to national spending on immunization as a measure of affordability. Costs were reported in 2023 international dollars (I$)

**Results:**

The total costs of designing, developing, and deploying eIR, eLMIS, or both were I$ 2.2, 6.4, 6.8, and 44.3 million in Guinea, Honduras, Rwanda, and Tanzania, respectively. Design costs were greatly affected by the degree of customization of the tool, whereas rollout costs were mostly driven by the costs of purchasing hardware and training health workers. Overall, the implementation of the electronic systems was associated with higher costs in Honduras (I$626 per facility, 95% CI 516-821) and Rwanda (I$399, 95% CI I$108-I$691), a cost reduction in Tanzania (−I$2539, 95% CI −I$4290 to −I$789) and no significant cost difference in Guinea. The percentage weight of the cost of managing data with the electronic systems over the total national immunization budgets was estimated at 0.7%, 7.7%, 3.3%, and 4.8% for Guinea, Honduras, Rwanda, and Tanzania, respectively.

**Conclusions:**

Digital health interventions such as eIR and eLMIS can potentially reduce costs and improve the efficiency of immunization data management and vaccine logistics in LMICs. However, the extent of cost savings depends on how effectively these digital systems replace traditional paper-based methods and the extent of their use in decision-making, especially at the facility level. Careful planning and investment are essential to unlocking the full economic potential of digital health in LMICs.

## Introduction

### Background

eHealth, short for “electronic health”, refers to the use of information and communication technologies in the health sector to enhance the delivery of health care services, improve patient outcomes, and facilitate the management and exchange of health-related information. It encompasses a wide range of digital tools, systems, and apps that are designed to support and enhance health care delivery [1]. In recent years, the development of digital health technology solutions has attracted considerable interest from the medical and public health communities, particularly in low- and middle-income countries (LMICs), where digital health, including mobile health solutions, is seen as capable of overcoming existing infrastructural and geographical barriers to widespread and equitable access to health services. The 2005 World Health Organization resolution on eHealth recognized the value of digital health interventions in achieving universal health coverage and meeting the sustainable development goals, urging ministries of health “to assess their use of digital technologies for health […] and to prioritize, as appropriate, the development, evaluation, implementation, scale-up, and greater use of digital technologies...” [2]. The strategic interest in digital health solutions was also confirmed in the World Health Organization Global Strategy for Digital Health 2020‐2025, which set 4 overall strategic goals to promote the institutionalization of digital health in the national health systems [3]. In a joint document released in 2015, the World Bank Group and the United States Agency for International Development acknowledged that countries could face challenges (time or skills) in producing quality health data and statistics, and this constitutes a key barrier to building stronger health information systems and called on governments, donors, and multilateral institutions to harness the power of digital health innovations to improve the availability, quality, and use of data for health decision-making [4]. This, in turn, has led to substantial investments in a wide range of digital health solutions by national and international funders in LMICs.

Within National Immunization Programs (NIPs), several national and international initiatives have been launched aiming at implementing electronic immunization registries (eIRs) and electronic logistic management information systems (eLMIS) in LMICs [5-18]. eIRs are eHealth interventions used to capture, store, access, and share individual-level, longitudinal immunization information in digitized records. Compared to traditional paper-based registries, eIRs are expected to improve the capacity to identify spatiotemporal trends in vaccination coverage and dropout, inform resource allocation and program operations, and target quality improvement measures and statistics [19-23]. eLMIS are designed to manage and optimize the logistics and supply chain processes related to health commodities, including vaccines and other medical supplies. Their adoption is expected to improve end-to-end visibility and thereby change both how frontline workers place orders and how supervisors allocate stock across facilities, ultimately resulting in fewer stock-outs over time [24].

Despite sustained interest in eHealth technologies for immunization programs and their implementation in several LMICs, robust evidence of the impact of these systems both in terms of programmatic outcomes [6,19,24-26] and costs [12,27,28] is still scarce and mostly focused on a small number of countries. This study aimed to provide additional evidence on the costs of implementing eIR and eLMIS systems in 4 LMICs, Guinea, Honduras, Rwanda, and Tanzania, and to estimate their economic costs as compared to the previous paper-based registries. More specifically, the following aspects have been investigated: (1) the upfront financial expenditures at national level for the design, development, and implementation; (2) the routine economic costs of data management with eIR or eLMIS; (3) the incremental economic costs for data management using the eIR or eLMIS compared to paper-based registries and reporting systems; and (4) the affordability and sustainability of maintaining the electronic systems based on each country’s current expenditures on health. The presented results in this study are an integral part of a broader evaluation that uncovered both the programmatic and economic impact of implementing eHealth interventions in these 4 countries.

### Study Setting

Guinea, Honduras, Rwanda, and Tanzania have different data management systems in place, at different scales of implementation and stages of maturity. Tanzania is the only country that has implemented both an eIR and eLMIS, whereas Guinea has implemented an eLMIS, and Honduras and Rwanda have an eIR. At the time of the evaluation, all countries maintained in parallel both a paper-based system and the electronic systems implemented either nationwide or piloted in selected regions.

In Guinea, the current eLMIS was custom-developed from the open-source OpenLMIS (version 2), mirroring the existing paper-based logistics management system. The tool tracks 185 essential health products across nine programs, including 12 products used for routine immunization under the Expanded Program on Immunization (EPI). The eLMIS currently captures consumption and stock data at the facility level, while vaccine management activities, such as ordering vaccines, rely on separate tools and paper-based records. Honduras developed a bespoke hybrid system, where the electronic system is installed at most midlevel health facilities (HFs) at the municipal level, while lower-level HFs at the rural level still record data on paper. Paper data are subsequently transmitted by facility staff to either midlevel HFs or regional offices for digitization and upload to the eIR [29,30]. Rwanda implemented an eIR package within the District Health Information Software Version 2.0, the web-based platform that is used nationally in both public and private HFs to support the management of data across several programs in the country. Tanzania began developing a Generic Immunization Information System platform, locally known as the Tanzania Immunization Information System (TIIS), in 2014. After a pilot in Arusha faced significant implementation challenges, the system was abandoned. In response, a new eIR was developed and deployed in four regions by 2018, eventually expanding to 15 of the country’s 26 regions through a phased approach. Similarly, the eLMIS was introduced in 2015 with a stepped implementation, starting with a pretest in 7 regions, followed by gradual expansion to 15 regions the next year. By 2018, the system had been implemented nationwide across all districts and regions [26]. Table 1 summarizes the main characteristics of the digital health solutions implemented in each country.

**Table 1.** Digital health solutions for immunization and vaccine logistics data management implemented in each country.

	Guinea	Honduras	Rwanda	Tanzania
Known as	eSIGL	SINOVA	e-Tracker	TImR (eIR^a^), VIMS (eLMIS^b^)
Technical platform	OpenLMIS	Custom development	DHIS2^c^ eTracker	OpenIZ/SanteSuite (eIR); OpenLMIS (eLMIS)
Implementation started	2018	2012	2019	2014 (eIR); 2015 (eLMIS)
Scale at the time of the evaluation	Nationwide	Nationwide	Nationwide	Nationwide (eLMIS); implemented in selected regions (eIR)
Level of implementation	At regional, prefectural, and communal health directorates and selected HFs^d^ (approximately 55% of HFs)	Hybrid approach. eIR used at regional and midlevel HFs. Lower-level facilities continue using paper forms	At the central and HF level. Not implemented at the regional level	Down to district level (eLMIS); down to facility level (eIR)
Implementation characteristics	A dual paper-electronic reporting system is used, with electronic data entered at the facility level (if eLMIS is implemented) or at the prefectural level	Electronic data are back-entered at midlevel primary care facilities or regional offices, while lower-level to higher-level HFs report using paper. Paper systems still used in parallel at all levels	Used variably at the facility level alongside a parallel paper process^e^	Variable implementation across regions, either fully electronic; parallel paper and electronic systems; or paper-only

a eIR: electronic immunization registry.

b eLMIS: electronic logistic management information system.

c DHIS2: District Health Information Software Version 2.0.

d HF: health facility.

e In Rwanda, the system was subsequently scaled up and paper-based registries were phased out nationwide.

## Methods

### Data Sources

A mix of primary and secondary data sources was used for collecting the necessary data for the analysis. In all countries, expenditure data for the design, development, and implementation of the tools were collected directly from the implementers and the country offices of the NIP. Primary survey data were collected to estimate the economic costs of immunization information systems with and without the use of electronic tools. Survey data were collected at different administrative levels, including HFs, district offices, and regional offices. In all countries, a purposive sampling of regions or provinces, districts, and HFs was adopted to achieve a balanced sample of health centers with relevant characteristics. Criteria for selection included the implementation status of the digital solutions, time from the first implementation, and geographical distribution and type of HFs. Table 2 reports the number of HFs, district health offices, and regional offices where primary data for the cost analysis were collected, whereas details of the criteria used in each country are reported in the Multimedia Appendix 1. Primary data were collected in October/November 2021 in Tanzania, February/March 2022 in Rwanda, April 2022 in Guinea, and September 2022 in Honduras. Questionnaires were distributed using portable electronic devices with Open Data Kit software and uploaded to central servers via the Kobo Collect app.

**Table 2.** Sample size for primary data collection in each country by administrative level.

	Guinea	Honduras	Rwanda	Tanzania
Health facility	43	80	24	61
District health office	7	—^a^	12	30
Regional health office	—	8	—	10
Total	50	88	36	101

a Not applicable.

### Data Analysis

The methodology adopted for cost analysis varied across different dimensions investigated in this study. We outline below the methods used for each of the four economic aspects mentioned above.

#### National Expenditures for Design, Development, and Implementation

The perspective used for the analysis of financial expenditures was that of a “third-party payer,” including the expenditures from external funders (eg, international organizations or private funders) and domestic funders (eg, national or subnational authorities). All data were retrospectively collected from the implementers of the tools and NIP offices in all countries. A descriptive analysis was conducted, classifying the financial expenditures into expenditures for the design and development of the tools and the expenditures for the rollout in the country. The costs considered included those for purchases of goods and services, such as for equipment, internet bundles, or the development of the digital health system, transport costs, training materials, and other direct costs.

In-kind contributions from local governments during the implementation of the tools (ie, in terms of government staff time spent for management, coordination, and operational activities, as well as goods and infrastructure made available to the implementation team) were only partially available for Honduras, Rwanda, and Guinea and not available for Tanzania. For Honduras, despite the nationwide implementation, detailed expenditure data were available only for the two regions of the pilot implementation of their eIR between 2012 and 2013. Therefore, to estimate the implementation cost for the whole country, the cost of the pilot phase was used to extrapolate the cost of implementing the eIR to the other 18 health regions (Multimedia Appendix 1). For Tanzania, the rollout costs also included the costs related to the development and pilot of the legacy eIR, the TIIS, which was later shelved and replaced by the current tool. The costs associated with designing, developing, and piloting the TIIS were sourced from a previous study by Muvundura et al [27]. In Guinea, since the eLMIS was developed to support logistics management for 9 programs, the proportion of vaccines managed through the eLMIS, relative to the total number of items handled by the system, was used as the cost driver to allocate the share of development costs attributable to the EPI. This proportion was estimated at 6.5%, based on data from the eLMIS itself.

#### Routine Economic Costs and Cost Impact of Implementing the Electronic Systems

An activity-based costing approach was used for the calculation of routine economic costs based on primary data collected. The activity-based costing approach consisted of identifying a series of activities performed by the staff of HFs, district, and regional offices and then tracing direct and indirect costs to these activities [31]. The activities considered were limited to those related to the management of immunization and vaccine stock data and were predefined based on a literature review and an iterative consultation process with experts in electronic immunization systems (Table 3).

**Table 3.** Activities defined to estimate the routine economic costs of the electronic tools using the activity-based costing approach.

Activity^a^	Description	System for which the activity is relevant
Vaccination registration	Data entry of vaccinee and vaccination session details regarding a new child registration	eIR^b^
Defaulter identification	Review of registries to identify children who missed appointments, making a list of defaulters	eIR
Defaulter contacting	Contacting defaulters to remind caregivers of missed vaccinations	eIR
Organizing outreach sessions	Preparing for the delivery of immunization in outreach settings	eIR
Delivery of outreach sessions	Time and resources spent on the delivery of immunization services outside the HF^c^	eIR
Vaccine quality control or monitoring	Physical counting, recording, and checking of closed or open vaccine vials for expiry dates or temperature excursions	eLMIS^d^
Cold chain monitoring	Data entry of refrigerator or freezer temperatures	eLMIS
Determining quantities of vaccine to order	Data mining and information extraction from dispensing or vaccine use and storage systems, and processing it to prepare the next order	eLMIS
Identifying performance gaps	Reviewing data to find performance gaps (such as HFs not being on track for coverage goals)	eIR and eLMIS
Report generation	Time spent to search for and record data that will be included in the regular reports on immunization services and stock management	eIR and eLMIS
Report transportation	Physical transport of weekly or monthly reports to a higher administrative level for submission	eIR and eLMIS
Refresher trainings	Recurrent training provided to HF staff on recording and reporting of immunization data, whether on paper or electronically	eIR and eLMIS
Technical or administrative support visits (supervision)	Recurring visits from higher health system administrative levels for supportive supervision and technical assistance in immunization service delivery	eIR and eLMIS
Maintenance	Time and resources spent on maintenance of systems and tools used in information management (eg, computers and printers)	eIR and eLMIS
Emergency vaccine replenishment	Time and resources spent on emergency stock replenishment from the distribution center in the event of a local stock-out	eIR and eLMIS
Printing	Printing of paper records, registries, tally sheets, reports, etc	eIR and eLMIS

a In each country, based on discussions with the EPI offices and local research partners, the description of activities and their inclusion in the questionnaires were further refined to better fit the characteristics of the tools used, the country-specificities, and the administrative level at which the survey was distributed.

b eIR: electronic immunization registry.

c HF: health facility.

d eLMIS: electronic logistic management information system.

In the questionnaires, respondents were asked to provide estimates of the number of staff and the amount of time spent on each of the defined activities, as well as other costs incurred for equipment, consumables, and services that were directly attributable to that activity. To estimate annual costs, information was also collected on the average frequency at which each activity was performed. Additional information was collected on printing and IT maintenance costs, which were shared across all the aforementioned activities. For each activity, mean costs were calculated together with 95% CIs. Answers to the questionnaires were checked both within and between questions to ensure internal consistency in the answers given by each respondent and to identify outliers or implausible values by reviewing distributions of values for each answer. When possible, inconsistent or implausible answers were corrected based on the qualitative comments given by respondents. Alternatively, a request for data validation was sent to the local research team, who verified with the data collectors and either confirmed the original values or provided an adjusted value. Answers that were considered by the research team as inconsistent or implausible and that could not be validated with the approach described above were considered as missing values.

Cost analysis was done individually for each activity described in Table 3 after disregarding missing values for which no imputation techniques were used. Staff time was converted to a monetary value using national reference salaries for health staff published in the Official Gazette or estimates on salary ranges directly reported by respondents during the interview (see Multimedia Appendix 1 for details). The cost per minute of staff was then calculated considering a monthly practical capacity equal to 20 days per month and 8 hours a day, and assuming a 20% reduction in capacity to account for sick leave, training, and breaks or leave. Unit costs for consumables and equipment were obtained from the NIP offices or local research partners. All IT equipment costs were then annuitized, assuming a useful life of 5 years. Indirect costs, such as utilities (electricity and internet), as well as maintenance and general costs of the health centers, were available to different extents in the 4 countries (Multimedia Appendix 1). Both shared and indirect costs were apportioned to the different activities described in Table 3, using as a cost driver the time spent for each activity over the total time spent on all activities. The costs of performing immunization and vaccine stock management activities were reported as the total average annual cost per HF. The costs at the district and regional levels were apportioned to each HF in the sample based on the total number of HFs delivering immunization services under the administration of the respective districts or regions. Given the different levels of digital tools’ implementation in the 4 countries, different approaches were used to estimate the cost impact of using electronic tools compared to paper-only systems. In Tanzania and Guinea, where implementation had not been achieved nationwide, the cost impact was estimated via an unadjusted cross-sectional comparison between HFs using and not using the electronic tools. In these countries, the estimated cost impact was calculated as the difference between the average economic costs of facilities using digital tools and those relying solely on paper-based systems. In Honduras and Rwanda, where the electronic tools had been implemented nationwide at the time of the evaluation, a before-and-after analysis was conducted using survey responses on resource use (staff time, consumables, equipment, etc) before and after the implementation of the electronic tools. All cost estimates were adjusted to 2023 real values using the World Bank gross domestic product deflator index and converted to international dollars (I$) using the 2023 World Bank’s Purchasing Power Parity conversion factor (I$1 equal to 11.1 Honduran Lempiras, 345.1 Rwandan Francs, 725.8 Tanzanian Shillings, and 2992.2 Guinean Francs). Analysis was done using Excel (Microsoft Corp) and R Studio (version 4.2; Posit). We used I$ instead of US $ to ensure consistent and meaningful economic comparisons across countries by accounting for differences in local prices and purchasing power. I$ serves as a standardized measure, reflecting the equivalent value of goods and services that a dollar can purchase in each country. This approach ensured that I$1 had the same purchasing power across all 4 countries considered in this study.

#### Affordability of Current Information Systems

In all countries, we estimated the overall cost of the immunization and logistics information systems at the national level. The projected costs reflected the national-level economic costs of current immunization and logistics information systems, given the current scale and level of implementation of digital tools in the country.

The number of HFs delivering immunization services was derived from official sources, including the 2022 National Register of Health Producing Units in Honduras, the Health Management Information System in Rwanda, the Health Facility Registry portal of the Ministry of Health in Tanzania, and the humanitarian data exchange by the United Nations Office for the Coordination of Humanitarian Affairs in Guinea.

In Rwanda, the cost of using the eIR at the national level was estimated by multiplying the projected cost per HF by the number of facilities providing immunization services in the country. A similar approach was used in Guinea, but accounting for the partial implementation of the eLMIS across facilities. Therefore, the cost of logistics information management at the national level was calculated by multiplying the costs estimated at the facility level with and without the eLMIS by the respective numbers at the national level, that is, 253 health centers where the eLMIS was introduced and 191 health centers that were still using the paper LMIS. In Tanzania, costs at scale were estimated based on the current implementation status across regions, with 15 regions having adopted both the eIR and eLMIS, and 11 having adopted only the eLMIS while still using paper-based immunization registries. For each region included in the sample, costs were calculated by multiplying the average cost per HF estimated for the region by the number of facilities providing immunization services. Costs for the remaining unsampled regions were assumed to be equal to the average costs observed in the sampled regions, grouped by implementation status. In Honduras, where a master list of health care facilities and their characteristics was available at the national level, the total cost was estimated using a regression-based approach. The average cost per facility was calculated using a generalized linear model with a gamma distribution and a log-link function. In the model, explanatory variables included the type of health care facility (midlevel primary care centers or lower-level facilities), the degree of management autonomy (centralized vs decentralized facilities), and a categorical variable that classified facilities based on how immunization data are digitized and transmitted to higher administrative levels. The regression results were then applied to the master list of Honduran facilities to predict national costs (Multimedia Appendix 1). A measure of sustainability was then considered by calculating the percentage weight of the yearly economic costs of using the electronic tools at the national level over the total government spending on immunization. Government spending was estimated using the cost of immunization per surviving infant estimated by Ikilezi et al [32] and the surviving infant population, which was calculated using live births and infant mortality data from Global Burden of Disease 2021. Spending on immunization in 2023 was estimated at US $16.2, US $41.8, US $11.6, and US $133.2 million, respectively, for Guinea, Honduras, Rwanda, and Tanzania (Multimedia Appendix 1).

### Ethical Considerations

The evaluation protocol and data collection instruments received ethical approval under the procedures set by the Tanzania Commission for Science and Technology in Tanzania, the Rwanda National Ethics Committee in Rwanda, the National Health Research Ethics Committee in Guinea, and the Pan American Health Organization Ethics Review Committee for Honduras. An informed consent form was signed by all respondents to the interviews and included explanations on the purpose of the study, procedure, benefits, risks, confidentiality, voluntary participation, and opting out. No compensation was provided to participants of the study. All data used in this study were anonymized to ensure the protection of participants’ identities. The anonymized data have been securely stored on a protected server, with access strictly limited to the authors responsible for conducting the analyses.

## Results

### National Expenditures for Design, Development, and Implementation

The upfront financial expenditures for the design, development, and rollout of the systems were mostly borne by external donors in all 4 countries (Table 4). Rollout costs were mostly driven by the costs of purchasing hardware and training of health workers, which accounted for 63%, 61%, 93%, and 48% of the total implementation costs for Guinea, Honduras, Rwanda, and Tanzania, respectively. In Tanzania, this cost also included the learning costs of the previously shelved digital tool for about I$ 1.6 million. The costs per HF relative to the design, development, and rollout are reported in Table 4.

**Table 4.** Financial expenditures for the development and rollout of the electronic systems in each country.

Country	System	Design and development, I$^a^	Rollout, I$	Total, I$
Guinea	eLMIS^b^	815,403	1,362,051	2,177,454
Honduras	eIR^c^	39,720	6,317,951	6,357,671
Rwanda	eIR	455,705	6,329,362	6,785,067
Tanzania	eIR/eLMIS	5,331,975/5,141,000	29,243,747/4,628,075	44,344,796

a I$: international dollars.

b eLMIS: electronic logistic management information system.

c eIR: electronic immunization registry.

The cost of tool design and development appeared to be linked to the characteristics of the system developed. Costs were higher in Tanzania and Guinea, where highly customized eIR and eLMIS tools were developed with technical assistance from external partners.

In contrast, Rwanda opted for an off-the-shelf eIR, the District Health Information Software Version 2.0, with only minor customizations, which was rolled out nationwide under the responsibility of the Ministry of Health. Similarly, Honduras developed a customized eIR by appointing an external national IT consultant for the task. However, the reported costs may not reflect the full expenses incurred in developing the system, as interviews with EPI members revealed that the development process underwent several iterations and changes that were not adequately planned for at the outset. As a result, many of these adjustments were carried out by the IT consultant without appropriate compensation. The observed low development costs in Honduras may therefore be attributed to the software being developed locally and an underestimation of the development effort, despite the tool being entirely newly developed.

### Routine Economic Costs and Cost Impact of Implementing the Electronic Systems

The routine economic costs of the electronic systems and the cost impact of using digital tools compared to performing data management activities using paper tools are summarized in Table 5. Labor costs accounted for the highest share of costs in all countries, comprising 49%, 72.1%, 85%, and 59% of the economic costs in Guinea, Honduras, Rwanda, and Tanzania, respectively. The costliest activities varied depending on the system used (ie, eIR, eLMIS, or both) and implementation setting (Multimedia Appendix 1). Specifically, the organization of outreach immunization sessions in Tanzania, report generation and transportation in Guinea, and child registration in Honduras and Rwanda were identified as the costliest activities.

**Table 5.** The annual economic costs of managing immunization or stock data management activities and the cost impact compared to paper-only registries.

Country	System	Annual economic cost^a^ of users of electronic systems (I$^b^), Mean (95% CI)	Cost impact per HF^c^ compared to nonusers of electronic systems (I$), 95% CI
Guinea	eLMIS^d^	857 (240 to 1493)^e^	−45.9 (−225 to 130)
Honduras	eIR^f^	6250 (5561 to 6938)^g^	626 (516 to 821)
Rwanda	eIR	1737 (1502 to 1974)	399 (108 to 691)
Tanzania	eIR+eLMIS	5741 (4542 to 6936)	−2539 (−4290 to −789)

a Cost of performing immunization or vaccine stock data management activities.

b I$: international dollars.

c HF: health facility.

d eLMIS: electronic logistic management information system.

e The annual economic cost in Guinea considered only the LMIS/eLMIS process and excluded the National Immunization Program legacy information flow, thus representing an incremental cost to the latter.

f eIR: electronic immunization registry.

g Honduras implemented a hybrid system, with the eIR installed only at midlevel facilities, while lower-level facilities transmit immunization data on paper to higher-level facilities for digitization. The reported cost represents the average across both types of facilities included in the sample.

Overall, the implementation of the electronic systems was associated with higher costs in Rwanda and Honduras, a cost reduction in Tanzania, and no significant cost difference in Guinea (Table 5). In all three countries where an eIR was implemented, the use of paper forms to record immunization data at the vaccination point was maintained in parallel with the electronic systems. This duplication of work was associated with an increase in costs for Honduras (+I$1268 per facility per year, 95% CI I$958-I$1468) and Rwanda (+I$336, 95% CI I$135-I$535), but not for Tanzania, where the activity of registering children with eIR was associated with a reduction in costs of approximately I$416 (95% CI −I$1030 to I$196) per year per HF. This reduction was linked to reduced staff costs, as less time was spent performing the activity by lower-paid workers. However, there was considerable variability across facilities, and the association between the cost reduction in Tanzania and the implementation of the tools, based on the qualitative comments by respondents, was not clearly identifiable. No other significant cost impacts were found for any other activity in Rwanda and Honduras, whereas in Tanzania, the integrated implementation of the eIR and eLMIS was also associated with a reduction in the costs for report generation and transportation (−I$493 and −I$347, respectively), vaccine ordering (−I$61), and identification of performance gaps (−I$422) and an increase in the costs for cold chain monitoring and supervision (I$373 and I$266, respectively; Multimedia Appendix 1).

### Affordability of Digital Tools

The extrapolated cost of managing immunization and vaccine logistics data using the electronic systems at the national level was equal to I$0.3 million, I$8 million, I$1.3 million, and I$21.1 million in Guinea, Honduras, Rwanda, and Tanzania, respectively. These costs reflect the situation at the time of the evaluation in Honduras and Rwanda, where parallel paper systems were in place, and a projection in the case of a national scale-up in Tanzania and Guinea, where implementation was only partial. In the case of Tanzania, scaling up the use of the eIR and eLMIS to the national level was estimated to generate savings of I$14.8 million per year compared to the current situation. The percentage weight of the cost of managing data with the electronic systems over the total national immunization budgets was estimated at 0.7%, 7.7%, 3.3%, and 4.8% for Guinea, Honduras, Rwanda, and Tanzania.

## Discussion

### Main Findings

We estimated the initial financial expenditures of implementing eIR and eLMIS and found that the upfront investment for the design, development, and rollout of the systems was mostly covered by external donors and was driven by hardware and training costs. In terms of their design and development, notably higher costs were observed for the bespoke or highly customized tools in Tanzania (I$10.7 million combined), while considerably lower costs were observed for Rwanda and Guinea, where off-the-shelf solutions were used. Overall, Guinea incurred the smallest upfront costs per HF for the implementation of the eLMIS, which was due to the implementation and use of the same eLMIS as for other health programs (eg, malaria or tuberculosis control programs), allowing significant economies of scope to be achieved. The total annual economic costs of managing immunization data using eLMIS and eIR represent between 1.1% and 8.6% of the total immunization expenditure.

There was mixed evidence on the cost impact of introducing these systems. Compared to the use of paper registries alone, the cost of managing immunization and vaccine stock data with the electronic systems was found to be higher in Honduras and Rwanda, lower in Tanzania, and negligible in Guinea. Notably, in 3 of the 4 countries, the implementation of a new process, inclusive of electronic systems, was in addition to the existing paper-based processes that remained in place with some adjustments. This duplication of processes resulted in higher costs for data recording compared to the paper registries, with the only exception of Tanzania. In addition, the extent to which these higher costs were offset by savings in activities that would benefit from more readily available electronic immunization data (eg, reporting and transport, planning routine activities at the facility level, or performance management) was linked to whether the electronic systems were used for decision-making, particularly at the HF level, which, in fact, was rarely the case. For example, the cost benefits of generating and transporting reports using electronic data would be immediately apparent. However, only in Tanzania did respondents clearly state that the introduction of the electronic systems had made this task easier, although infrastructural problems such as limited connectivity and an unstable electricity grid remained. The fact that paper registries were considered the primary source of information in almost all countries may have affected the overall quality and completeness of the electronic data and hampered the use of the systems to inform immunization activities, resulting in lower cost savings. The maintenance of a dual system has been specifically identified as a source of dissatisfaction among health workers in previous studies [33,34].

Overall, results in all 4 countries suggest that if the electronic systems were used as the primary source of information, managing immunization and vaccine stock data by electronic means would be cheaper than with paper-based systems, even if maintaining the latter exclusively as backup. This finding is in line with a simulation by Dolan et al [22] in Kenya. However, achieving such savings would be possible only if investments were dedicated to strengthening the ecosystem in which eIR and eLMIS are rolled out (ie, infrastructure and local capacity), enabling their sustainability. In another survey-based micro-costing study in the Arusha region of Tanzania, Mvundura et al [27] reported cost savings of US $10,236 (equivalent to I$39,651) per facility per year, much higher than the savings we estimated. As in this study, the savings were driven by reduced staff time for delivery of fixed and outreach immunization services, logistics and stock management, and data reporting. However, the authors did not clarify which activity contributed most to the reported cost savings, making a more detailed comparison difficult. In the same study, the authors estimated the savings associated with the introduction of an eIR in Zambia to be US $628 (equivalent to I$3356).

This study has provided additional evidence on the costs of designing, implementing, and operating eIR and eLMIS in 4 LMICs. The information gathered may be useful in understanding the likely impact of these eHealth interventions on immunization costs and their key determinants, helping to prioritize national and international funding in this area.

### Limitations

This study has several limitations. The secondary data collected to estimate the design and development costs of the electronic systems were of variable quality and availability between countries, which may explain some of the variability observed. In addition, certain relevant costs, such as in-kind contributions from local governments, were not available in all countries. In addition, this study was observational in nature, and therefore, estimating the cost impact of implementing the electronic systems versus the previous paper-based systems may be subject to bias. Primary data were collected using a purposive sampling approach. This method was chosen for practical reasons and to ensure the inclusion of facilities with diverse characteristics that could influence both costs and the programmatic impact of digital tools. While this approach allowed for the targeted exploration of key factors, it may have limited the generalizability of the findings and introduced potential bias in the calculation of aggregated national-level costs. Depending on the extent of implementation, the impact was estimated either by making a pre-post comparison at the facility level or by making an unadjusted comparison between HFs that had implemented the systems and those that had not. Both approaches can be subject to several biases, including the effect of other fixed or time-varying missing variables or reporting or recall errors. Studies prospectively collecting cost and programmatic data or using experimental or quasi-experimental approaches would provide more robust evidence on the impact of digital tools on immunization. Finally, another limitation of this study is the potential for residual data errors despite our meticulous data curation process. While outlier values and potential inconsistencies were cross-verified with data collectors, and where feasible, with respondents, there remains a possibility of inadvertently discarding true values or including erroneous or implausible data points. These errors may have introduced a degree of measurement error, potentially affecting the robustness of our findings.

### Conclusions

Digital health solutions have the potential to bring about significant benefits in LMICs by improving health access, enhancing productivity, streamlining data management, minimizing paperwork, and optimizing supply chain management. Furthermore, improved data collection and analysis through digital health systems can inform evidence-based health policies, leading to more efficient resource allocation. However, this study clearly shows that the economic impact of digital health solutions greatly depends on factors such as infrastructure, implementation, and the extent to which these technologies are integrated into existing health care systems. Careful planning and investment are essential to realizing the full economic potential of digital health in LMICs.

## Supplementary material

10.2196/62746 Multimedia Appendix 1 Additional materials and detailed results.

## Abbreviations

eIR: electronic immunization registry
eLMIS: electronic logistic management information systems
EPI: Expanded Program on Immunization
HF: health facility
I$: international dollar
LMIC: low- and middle-income country
NIP: National Immunization Program
TIIS: Tanzania Immunization Information System
