# Supplementary material for: The Costs of Digital Health Interventions to Improve Immunization Data in Low- and Middle-Income Countries: Multicountry Mixed Methods Study
Source: J Med Internet Res. 2025 Aug 18;27:e62746. doi: 10.2196/62746 (PMC12360727; doi:10.2196/62746)
Supplement: Multimedia Appendix 1 [file jmir-v27-e62746-s001.docx]

## The Costs of Digital Health Interventions to Improve Immunization Data in Low- and Middle-Income Countries: aAMulti-Ccountry Mixed Methods Study

# Additional materials and detailed results.

## Sampling

Table summarizing the main criteria used for sampling

|  | Rationale | Country examples/specificities |
| --- | --- | --- |
| Implementation status | To achieve a balanced sample of HFs using (intervention group) and not using the electronic tool(s) (control group), where not implemented nationwide. | In Rwanda, the eIR was rolled out nationally, so assessment of use for sampling was based on frequency of use for reporting of immunization data. Guinea and Honduras first rolled out processes and tools nationally, followed by a partial (either at center or regional level) implementation of the digital components of the systems. |
| Time from first implementation | HFs were selected in the intervention group which had been using the tools for a sufficient time to ensure that an effect/ impact could be observed. | Regions in Tanzania which had introduced the tools first and had already been evaluated were selected for comparison. In Guinea HFs which had received training at least 3 months before the data collection were selected to ensure that sufficient time had elapsed between the introduction of the tool and the evaluation. |
| Health facility geographical distribution | Different regions and districts were selected to reflect the different state of implementation and conditions and to achieve a balanced mix of urban and rural HFs. | In Guinea only 4 rural HFs had introduced the system at the time of data collection, and this resulted in an over-sampling of urban centers. |
| Health facility type | To arrive at a representative sample HFs across selected districts and regions were selected to reflect different conditions of use of the systems (access to electricity and internet, availability of computers, number of personnel and training) | Health facilities selected include dispensaries, health centers and hospitals in Tanzania; public and private HFs in Rwanda; communal medical centers and health centers in Guinea; Integrated Health Centers (CIS) and Primary Health Care Units (UAPS) in Honduras.  Access limitations and of security issues were considered in Honduras and Guinea. |
| Other indicators | Other programmatic characteristics were considered to ensure that facilities with diverse performance and needs were included. | Other indicators included the size of HF catchment population (Rwanda and Honduras); number of doses delivered per month (Tanzania); DTP3 coverage (Guinea and Honduras); DTP1-3 and DTP1 to MR1 drop-out rates (Rwanda). |

## Financial expenditures – Sources of data and assumptions

#### Honduras

Detailed expenditure data was available from the EPI offices who implemented the system only for the pilot implementation of SINOVA in the two regions of Francisco Morazán and Comayagua where SINOVA has been rolled out between 2012 and 2013. For these two regions an analysis of the implementation costs by type of expenditure and input was conducted using financial documents which were retrieved from the country’s EPI office. These documents report the expenditures for different activities related to the implementation of SINOVA in the two regions, with details on the amounts of resources consumed, the purpose of the expenditure, and the source of funding, whether domestic, or external, mostly from the World Health Organization / Pan-American Health Organization (WHO/PAHO), and Gavi Vaccine Alliance. Notably, during the period of the implementation of the pilot, purchases of only 7 computers were reported. These low quantities may be explained by the fact that originally, data entry in the SINOVA software was configured to be done at the regional level, not at the municipal level. It was only decided later to configure data entry at HF level and, therefore, to also provide HFs with computer with SINOVA software installed. To account for this additional expenditure, the acquisition cost of computers was added to the original implementation costs, if all integrated health centers (Centro Integrado de Salud – CIS) in the SINOVA regions received a PC for data entry (personal communication from head statistician at Francisco Morazán). Specifically, 21 and 27 computers were assumed to be purchased for Comayagua and Francisco Morazán respectively as part of the implementation costs at a unit acquisition cost of USD 2,000 each (personal communication from the EPI office). To estimate the implementation cost for the whole country, the cost of the pilot phase was used to extrapolate the cost of implementing SINOVA to the other 18 health regions. We excluded in this extrapolation the upfront costs incurred for software development and purchasing of equipment for the central level, which were assumed to be incurred only once for all health regions. The cost for equipment and supplies and the cost for IT hardware and software were assumed to be proportional to the number of CIS in the region, since only CIS received IT equipment and other supplies during the roll-out of SINOVA. Similarly, the costs for printing and servers were assumed to be a linear function of the total population under 1 year in each Region, whereas the cost of training and the cost for implementation assessment and supervision from the central level were assumed to be a linear function of the whole number of HFs providing immunization in the region, i.e., both CIS and UAPS. For each type of cost, the same share of government co-financing was assumed as in the two pilot regions.

#### Rwanda

Expenditure data were obtained from the Health Management Information Systems (HMIS) Rwanda and the the nation's central health implementation agency, Rwanda Biomedical Center (RBC). Available costs included costs for IT equipment (tablets and desktop PCs and modems), trainings, and software development. While initially planned for the eIR, the investment in tablets in 2020 was redirected towards supporting the COVID-19 response which leveraged the DHIS2 platform for scheduling COVID-19 vaccinations and tracking their delivery through tablets in facilities which were different from the ones used for routine immunization. Therefore, despite constituting a part of the financial expenditures, the cost for tablets was not considered in the costs analysis, which instead included the annuitized cost of one computer per HF. Training was delivered to a total of 1,738 HWs through training of trainer (TOT) and cascade trainings at 3 administrative levels (i.e., central, DH and HF level) during a 3-day period. The trained workforce included officers from all levels down to community, data managers, district VPDP supervisors as well as nurses, vaccinators and the heads of HFs. No information on in-kind contributions from the local government were available (e.g., in terms of government staff time spent for management, coordination and operational activities, as well as goods and infrastructure made available to the implementation team) and, therefore, were not considered in this analysis.

#### ****Tanzania****

The costs incurred to implement the VIMS and TImR were based on data obtained from the following sources: the immunization and vaccines development (IVD) Program of Tanzania which owns both systems; PATH and JSI (i.e., the organizations which supported the development and implementation of the systems); and Santé Suite Inc. which provided technical assistance in the development of the TImR. No expenditures were directly available concerning the Better Immunization Data (BID) staff who were overseeing implementation during the initial roll-out of the system in Arusha, Tanga and Kilimanjaro, and for the TImR implementation in the Dodoma region in the year 2018. As such, this information was taken from published literature. Specifically, Mvundura et al.^[[1]](#footnote-2)^ (2019) estimated BID staff cost at USD 1.6 million for the three regions, whereas the costs for the TImR implementation in Dodoma were reported to be USD 89,795 in a costing study by Mott MacDonald (2019)^[[2]](#footnote-3)^.

Detailed accounting information and expenditure reports were not able to be obtained during data collection. As such the analysis of financial expenditures is based on available budgets, assuming that these were consumed entirely for the budgeted purposes and thus the budgets reflect the actual expenditures incurred. In addition, no information on in-kind contributions from the local government were available (e.g., in terms of government staff time spent for management, coordination and operational activities, as well as goods and infrastructure made available to the implementation team) and, therefore, were not considered in this analysis.

#### Guinea

Financial expenditure data for the eLMIS design and development, as well as for the implementation to date were obtained from Chemonics (a private international development firm based in Washington DC working often as recipient of USAID fundings). This data reflects the expenditures incurred by the Global Fund and Chemonics in 2018, as the two implementers of the project. These two organizations were equally responsible for financing the design and development of the system and its deployment, with domestic contributions from the Government of Guinea. The eLMIS in Guinea was implemented at the outset for 9 programmes, including the Expanded Programme for Immunization. Indirect costs are then shared across these programmes. To calculate the share of costs to the EPI the number of tracer products included in the eLMIS was used as cost-driver. Twelve immunization products are included in the list of tracer products: 7 vaccines including BCG, bOPV, Pentavalent, IPV, Measles, Meningitis-A, and Tetanus-Diphtheria adults (Td), as well as other ancillary items, such as syringes. These 12 products represent 6.5% of the total number of items managed through the eLMIS system for nine programs (Guinea Technical Committee, 2021).

#### Staff and other unit costs

##### Guinea

Staff time was converted into monetary values using the national reference salaries for health staff published in a 2019 report from Appaix, Bah, & Maritano, 2019^[[3]](#footnote-4)^. Other information on unit costs for consumables were retrieved from the technical committee formed to provide a directive counterpart to the researchers deployed in the field. The technical committee was composed of five executive officers in the Ministry of Health with different positions and competences, including the heading up of the integrated logistics unit, and management of the vaccination programs and the “strategies and development unit”. Electricity and Internet costs were provided by the Ministry of Health and Chemonics respectively. Unit costs for IT equipment, including laptops, desktop PCs and servers, were provided by Chemonics, based on their purchasing costs at the time of the implementation.

| Cost Item | Health Center type | Cost (USD) | Source |
| --- | --- | --- | --- |
| Healthcare Staff (salary per month) | | | |
| Healthcare worker | Health Centre | 2,097 | (28) |
|  | Community Medical Center | 2,246 |  |
| Pharmacist | Health Centre | 2,995 |  |
|  | Community Medical Center | 3,594 |  |
| Nurse | Health Centre | 2,546 |  |
|  | Community Medical Center | 2,696 |  |
| Consumables | | | |
| Cost of paper per page |  | 0.09 | Guinean technical committee |

Given that eLMIS is a tool shared across multiple health programs, all indirect costs, such as internet and electricity charges, were allocated to the vaccine management activities using staff time as a cost driver (i.e., by allocating a percentage of these costs equal to the time spent on each activity for vaccine management relative to the total available staff time of the health centers). The allocation factor applied for indirect costs was 6.5%, based on the number of vaccines over the total number of items managed by the eLMIS, as done with the apportioning of the initial financial expenditures.

##### Honduras

Average salaries of staff performing the activities were estimated using the information provided by the respondents on their role and monthly salary range. Specifically, for each reported salary range the mid-point was calculated as the average of the indicated lower and upper bound. Then, weighted averages of the salaries for each role were calculated using the frequency of each salary level in each role category as weight. The final table of salaries was then validated by the local research team and is reported below. Unit costs for IT equipment and other consumables (e.g., cost of printing) were communicated by the EPI staff, based on available financial documents during the implementation of the electronic systems.

| **Cost Item** | **Cost (USD)** | | **Source** |
| --- | --- | --- | --- |
| **Healthcare Staff (salary per month)** | | | |
| Director of HF | 31,333 | | Survey data |
| Doctor | 29,000 | |  |
| Professional Nurse | 24,045 | |  |
| Admin Officer | 18,000 | |  |
| Statistician | 18,000 | |  |
| Staff from central EPI office | 18,000 | |  |
| Auxiliary Nurse | 15,661 | |  |
| Driver | 15,661 | |  |
| Data clerk | 13,000 | |  |
| AGI Technician | 8,000 | |  |
| Student/Trainee | 2,500 | |  |
| **Consumables** | | | |
| Paper per page | 0.059 | | Personal communication, EPI office Honduras |
| **Equipment** | | | |
| Computers and Servers (one set) for regional offices | | 2,000 | Personal communication, EPI office Honduras |
| Printers for Regional Offices | | 800 |  |
| Computers for health facilities | | 900 |  |
| Printers for health facilities | | 300 |  |

Due to the unavailability of reliable accounting documents from Central level, Regional offices or health facilities, no overhead and indirect costs such as electricity, vehicles or space use were considered in addition to the direct or shared costs collected in the questionnaires (i.e., on printing and maintenance of IT equipment).

##### Rwanda

Staff time was converted to a monetary value using national reference salaries for healthcare staff.

| Cost Item | Unit cost (USD) | | Source | |
| --- | --- | --- | --- | --- |
| Healthcare Staff (salary per month) | | | | |
| Healthcare Staff (salary per month) | 421 | | Official Gazette, 2020 | |
| EPI Supervisor (District) | 612 | |  |  |
| M&E Officer (District) | 361 | |  |  |
| Nurse A0 | 292 | |  |  |
| Nurse A1 | 292 | |  |  |
| Vaccinator | 292 | |  |  |
| CHO-Community Health Officer | 292 | |  |  |
| Data Manager (District) | 361 | |  |  |
| Data Manager A1/A0 | 250 | |  |  |
| Accountant A1 | 250 | |  |  |
| Nurse A2 | 173 | |  |  |
| DH Technician | 250 | |  |  |
| Hired Company | 250 | |  |  |
| Assistant Head of Health Center | 250 | |  |  |
| **Consumables** | | | | |
| Cost of paper per page | 0.004 | | Provided by Rwandan research partner, CIIC-HIN | |
| **Equipment** | | | | |
| Computer | | 500 | | WHO CHOICE estimates |

In addition to the primary data collected, indirect and shared costs were obtained from secondary data sources. Specifically, expenditure data from 2018 and 2021 for the 24 HFs in the sample were obtained from the Rwanda Health Management Information System (RHMIS). Cost data for the following categories were available: (i) communication, telephone, and internet; (ii) purchase of non-medical equipment; (iii) maintenance and repair of infrastructure; and (iv) office supplies, printed materials, medical records. All indirect costs were apportioned to the activities in scope using staff time per activity as the cost driver (i.e., by allocating a percentage of these costs equal to the overall time dedicated to each activity over the total available time of all the personnel of the HFs). The latter was calculated using data on the number of employed staff per facility published in the official Gazette, and assuming a monthly practical capacity of each staff member equal to 20 days per month and 8 hours a day, and assuming a 20% reduction in capacity to account for sick leave, trainings and breaks/leave.

##### Tanzania

Staff time was converted to a monetary value using national reference salaries for healthcare staff, which provided lower and upper salary limits per staff profile. In the cost calculation the mean of these lower and upper boundaries were used

| Cost item | Unit cost (USD) | Source |
| --- | --- | --- |
| Healthcare Staff (salary per month) | | |
| Assistant clinical officer | 307 | National reference salaries, official gazette (2015) |
| Assistant DIVO / RIVO | 701 |  |
| Assistant medical officer | 701 |  |
| Clinical officer | 544 |  |
| Community Health Worker^a^ | 212 |  |
| DIVO / RIVO^b^ | 992 |  |
| Driver | 349 |  |
| Enrolled nurse | 416 |  |
| Environmental health officer | 212 |  |
| Facility in-charge^c^ | 768 |  |
| Medical Doctor | 992 |  |
| Nurse | 468 |  |
| Nurse attendant | 212 |  |
| Public health nurse | 468 |  |
| Registered nurse | 622 |  |
| Registered nurses with Degree | 701 |  |
| Registered nurses with Diploma | 544 |  |
| Surveillance coordinator | 307 |  |
| Technician | 348 |  |
| Vaccinator | 212 |  |
| Consumables | | |
| Cost of paper | 0.02 | Personal communication from local partners |
| Equipment | | |
| Tablet | 522 | Personal communication from local partners |

*DIVO: District Immunization and Vaccine Officer; Regional Immunization and Vaccine Officer, ^a^Assumed to be the same as nurse attendants; ^b^Assumed to be the same as medical doctors; ^c^Facility in charge is the person in charge of the hospital or health center and in the Hospitals and Health Centres they are Medical Doctor but at dispensary level we have Medical Doctor and Clinical officer as facility in charge.*

Data on shared cost pertaining to recurrent services, consumable and durable goods were directly elicited from the questionnaires or derived from the 2021 immunization budget of the Tanzania Immunization and Vaccine Development Program. These costs were then allocated across all activities using staff time as a cost driver and included printing and maintenance costs as well as costs of internet, and of distributing paper registries from central to lower administrative levels. Each facility was also assumed to be endowed with an electronic tablet, whose cost was apportioned across all reported activities using the same cost-driver. Other indirect and overhead costs (e.g., those related to facility costs on electricity or other maintenance costs) were not able to be obtained due to lack of reliable data sources and, thus, were excluded from the analysis.

#### Extrapolation of the economic costs of using eIR and eLMIS to national level

To calculate the total cost of using the electronic systems to national level, the calculated costs per HFs were applied to the overall number of HFs in the country. The number of HFs delivering immunization was derived from official sources such as the 2022 National Register of Health Producing Units (RUPS) in Honduras, the Health Management Information System (HMIS) in Rwanda and the Health Facility Registry (HFR) portal of the MoH (HFR portal, 2022) in Tanzania and the Ministry of Health, and the humanitarian data exchange by the United Nations office for the coordination of humanitarian affairs^[[4]](#footnote-5)^ in Guinea. The total number of HFs considered for the calculations is reported in the table below

| **Country** | **No of Health Centers providing immunization in the country** |
| --- | --- |
| Honduras | 1,237 of which 360 mid-level primary care centres (Centro integral de salud - CIS) and 28 polyclinics where the eIR was implemented and 849 low-level care centres (UAPS) where the former paper-based registries were maintained. |
| Rwanda | 505 health facilities |
| Tanzania | 5,497 delivering immunization, of which 3,768 in 15 Regions having the eIR+eLMIS implemented |
| Guinea | 444 health facilities, of which 253 with the eLMIS introduced alongside the previous paper-based system |

##### Guinea

The total costs currently incurred by the country to perform reporting activities on vaccine consumption and stock levels using the eLMIS has been calculated based on two assumptions: i) the sample is representative, from a costing perspective, of the 444 health centers in the country providing immunization services, and ii) all health centers where the eLMIS has been introduced to date are using the system. The calculation of the total cost of managing vaccine logistics data was then calculated by multiplying the costs estimated at facility level for users and non-users of the eLMIS by the respective numbers at national level, i.e., 253 health centres where the eLMIS was introduced and 191 health centres that were still using the paper LMIS. Additional costs incurred at the central level were considered when calculating the national operating costs of using the eLMIS. These included personnel costs (I$ 8,456 per year), internet costs (I$ 4,553), IT maintenance costs (I$ 1,115), data hosting (I$ 310) and Security licences (I$ 139)

##### Honduras

The total cost of managing immunization data for the whole country was calculated starting from the cost estimates per HF using a regression-based approach.  First, a generalized linear model using a Gamma distribution with a log-link was used to account for the typical features of cost data, consisting of only positive values and often skewed distributions.  Explanatory variables used included the type of HF (i.e., mid-level primary care centres  - *CIS* or lower- level HFs - UAPS); the centralized/non-centralized management; and the categorical variable that classifies HFs according to the way nominal immunization data is digitized and transmitted. Regarding the latter, 4 types of facilities were distinguished 1) HFs that directly digitize immunization data into the eIR and then send the electronic records to the higher administrative levels via email; 2) HFs that digitize the paper forms into the eIR software but then, due to lack of internet connection, physically transport the electronic records to the higher administrative level on a USB drive or other physical support; 3) HFs that transport paper forms to another HF to be digitized, due to lack of basic infrastructure, equipment, or human resources to perform this activity and 4) A residual group of HFs that neither digitize nor transport the paper forms to other HFs or to the higher administrative level, mainly due to lack of resources. Information on rural/urban HFs was not used as it was almost perfectly overlapping with the type of HF since mostly all CIS are urban HF and mostly all UAPS are rural HF. Individual regressions were conducted for each activity included in the Activity Based Costing surveys using as dependent variable the full cost of managing immunization data. For each regression, model selection was performed using a stepwise approach and choosing the best model based on the lowest Bayesian Information Criterion value. Second, the results of the regression models were then used to extrapolate the estimated cost to the whole set of HFs in Honduras by predicting costs estimates on a new dataset which was representative of the distribution of Honduran HFs. Specifically, for the prediction dataset, the distribution of types of HF providing immunization in Honduras (i.e., CIS, UAPS and Policlinics) was taken from the RUPS 2022. Notably, since only 4 Polyclinics were present in our sample, we assumed that the cost of managing immunization data in the 28 Policlinics providing immunization in Honduras was the same as in CIS and no distinction was made between these two types of HFs. The proportions of HFs for each level of use of SINOVA was then taken from the sample, assuming that such proportions were representative for the whole sampling frame.

##### Rwanda

the yearly recurrent cost of using the e-Tracker at a national scale was estimated based on the assumption that the sample of 24 HFs included in the evaluation was representative of the 505 HFs of the country delivering vaccination, and the annual operating cost of the e-Tracker. Cost at national level was then calculated by multiplying the cost per HF times the number of facilities in the country. Additional costs incurred at the central level were considered when calculating the national operating costs of using eIR. These included costs for refresher trainings (I$ 736,054 per year), and printing costs of paper forms (I$ 14,983).

##### Tanzania

The total annual cost for the entire country was estimated based on the mean costs per HF using VIMS+TImR or using VIMS+paper IR. For each region, the cost for immunization data management was calculated by multiplying the mean cost per HF in each region with the total number of HFs delivering immunization services in that region. Then the cost for the other regions was extrapolated assuming that: i) the mean cost of the 6 regions with VIMS+TImR was generalizable to the total of 15 regions that have implemented the system so far and ii) the mean cost for the 4 regions that still rely on paper for immunization data management but have the VIMS installed was generalizable to the 11 regions in which the TImR is yet to be introduced. Additional I$ 2M were considered to account for the costs incurred by the central level such as costs for electricity, internet, technical assistance and system improvements, replacement of hardware, and servers.

## Estimating Government spending on Immunization

Government spending on immunization was estimated using the expenditure estimates per surviving child from Ikilezi et al (2021)^[[5]](#footnote-6)^ and Global Burden of Disease (GBD) estimates on livebirths and infant mortality rates (GBD 2021).

### Input parameters used to estimate Governments spending on immunization

| **Variable** | **Value** | **Source** |
| --- | --- | --- |
| **Livebirths 2023 (Number)** |  |  |
| Guinea | 476,041 | GBD 2021 |
| Honduras | 212,299 |  |
| Rwanda | 366,489 |  |
| Tanzania | 1,884,018 |  |
| **Infant Mortality (rate x 100,000)** |  |  |
| Guinea | 5,748 | GBD 2021 |
| Honduras | 1,229 |  |
| Rwanda | 2,991 |  |
| Tanzania | 3,859 |  |
| **Government spending per surviving infant (2019) (USD)** |  |  |
| Guinea | 15 | Ikilezi et al. 2021 |
| Honduras | 94 |  |
| Rwanda | 14 |  |
| Tanzania | 31 |  |
| **Yearly growth rate of Government Spending (%)** |  |  |
| Guinea | 10% | Ikilezi et al. 2021 |
| Honduras | 7.7% |  |
| Rwanda | 10% |  |
| Tanzania | 10% |  |

Government spending for 2023 was estimated based on Ikilezi et al., assuming an annual increase of 10% in sub-Saharan African countries and 7.7% in Honduras. The estimates were adjusted for inflation to 2023 USD using the World Bank GDP deflator index. The resulting values were then converted to international dollars using country-specific Purchasing Power Parity conversion factors and compared to the total national-level spending on the digital tools and management of immunization and logistics information.

## Detailed Results by activities

### Operating costs of health facilities per year by activity and type of cost (2023 I$)

##### **Honduras**

| **Activity** | **Personnel cost, I$ (95% CrI)** | **Other direct costs, I$ (95% CrI)** | **Total, I$ (95% CrI)** |
| --- | --- | --- | --- |
| Child registration on paper (SINOVA-1) | 729 (539; 831) | 78 (32; 94) | 807 (580; 919) |
| Digitizing of SINOVA-1 forms | 681 (500; 907) | 212 (132; 229) | 917 (675; 1,135) |
| Organizing outreach sessions | 1,003 (738; 1,364) | 39 (12; 42) | 1,044 (766; 1,390) |
| Defaulter identification | 541 (307; 641) | 37 (12; 50) | 578 (324; 687) |
| Defaulter contacting | 1,273 (892; 1,419) | 59 (24; 78) | 1,337 (939; 1,478) |
| Identifying performance gaps | 609 (400; 765) | 21 (8; 23) | 632 (418; 782) |
| Generating and transmitting SINOVA-2 report | 412 (370; 424) | 342 (116; 813) | 763 (505; 1,231) |
| **Total** | **4,923 (4,167; 5,216)** | **788 (449; 1,162)** | **5,692 (4,805; 6,052)** |

##### Rwanda

| **Activity** | **Personnel cost, I$ (95% CrI)** | **Other direct costs, I$ (95% CrI)** | **Indirect costs, I$ (95% CrI)** | **Total costs, I$ (95% CrI)** |
| --- | --- | --- | --- | --- |
| Child registration | 660 (493; 827) | 33 (15; 35) | 98 (55; 140) | 791 (618; 964) |
| Defaulter identification | 128 (67; 189) | 6 (2; 6) | 24 (10; 37) | 157 (95; 220) |
| Organizing outreach sessions | 329 (206; 452) | 18 (8; 21) | 24 (15; 34) | 372 (248; 495) |
| Identifying performance gaps | 241 (168; 314) | 8 (4; 7) | 29 (17; 41) | 278 (203; 352) |
| Report generation | 116 (80; 152) | 5 (2; 5) | 20 (11; 28) | 141 (104; 178) |
| **Total** | **1,475 (1,244; 1,705)** | **69 (40; 65)** | **195 (147; 243)** | **1,739 (1,502; 1,975)** |

##### Tanzania

| **Activity** | **Personnel cost, I$ (95% CrI)** | **Other direct costs, I$ (95% CrI)** | **Total, I$ (95% CrI)** |
| --- | --- | --- | --- |
| Child registration | 1,225 (955; 1,495) | 46 (0; 73) | 1,271 (998; 1,544) |
| Organizing outreach sessions | 1,295 (696; 1,895) | 364 (25; 528) | 1,660 (1,009; 2,310) |
| Defaulter identification | 450 (261; 639) | 189 (0; 316) | 639 (386; 894) |
| Defaulter contacting | 542 (346; 738) | 166 (7; 195) | 708 (498; 920) |
| Cold Chain Monitoring | 369 (294; 444) | 95 (8; 116) | 464 (377; 550) |
| Vaccine Ordering | 196 (146; 246) | 68 (0; 86) | 264 (200; 328) |
| Vaccine Quality Monitoring | 489 (290; 687) | 172 (2; 269) | 661 (421; 900) |
| Refresher Trainings | 3 (2; 4) | 119 (7; 230) | 121 (10; 233) |
| Supervision | 25 (8; 41) | 70 (0; 166) | 95 (-4; 194) |
| Identifying performance gaps | 567 (347; 788) | 121 (4; 165) | 688 (456; 920) |
| Report generation | 565 (416; 713) | 153 (0; 192) | 717 (545; 889) |
| Report transportation | 64 (8; 120) | 401 (236; 478) | 465 (329; 600) |
| **Total** | **5,789 (1,525; 10,053)** | **1,963 (793; 2,088)** | **7,753 (3,423; 12,082)** |

##### Guinea

| **Activity** | **Personnel cost, I$ (95% CrI)** | **Other direct costs, I$ (95% CrI)** | **Indirect costs, I$ (95% CrI)** | **Total costs, I$ (95% CrI)** |
| --- | --- | --- | --- | --- |
| Report generation | 193 (0; 473) | 106 (4; 208) | 4 (2; 7) | 304 (0; 667) |
| Report transmission | 170 (15; 325) | 142 (51; 234) | 3 (1; 5) | 316 (126; 505) |
| Determining quantities of vaccine to be ordered | 50 (10; 90) | 43 (0; 113) | 5 (0; 10) | 98 (17; 178) |
| Monitoring of performance indicators | 12 (0; 24) | 66 (-8; 139) | 6 (-3; 15) | 83 (6; 161) |
| Supervision | 0 (0; 1) | 48 (0; 120) | 17 (1; 33) | 66 (0; 138) |
| **Total** | **426 (103; 748)** | **406 (221; 590)** | **35 (15; 54)** | **866 (240; 1,491)** |

1. Mvundura M, Di Giorgio L, Lymo D, Mwansa FD, Ngwegwe B, Werner L. The costs of developing, deploying and maintaining electronic immunisation registries in Tanzania and Zambia. BMJ Glob Health. 2019 Nov;4(6):e001904. [↑](#footnote-ref-2)
2. Mott MacDonald. (2019). Evaluation of the Better Immunization Data Initiative. https://bidinitiative.org/wp-content/uploads/MM_BID_Final-Evaluation_Synthesis_Report_FINAL_Revision-3_100919.pdf. Accessed on 17 October 2022. [↑](#footnote-ref-3)
3. Appaix O, Bah AA, Maritano M. Assistante Technique au « Projet d’Appui à la Santé en République de Guinée (PASA) » [Internet]. 2019. Report No.: Europeaid/135187/IH/SER/GN. Available from: https://portail.sante.gov.gn/wp-content/uploads/2022/01/PASA1-Rapp-Etude-couts-Guin%C3%A9e-VF-10.06.019.pdf [↑](#footnote-ref-4)
4. [https://data.humdata.org/dataset/structures_de_sante_guinee_vf/resource/3d5e7ab6-5164-457d-bcc8-3cc1aa9b01aa](https://eur01.safelinks.protection.outlook.com/?url=https%3A%2F%2Furldefense.com%2Fv3%2F__https%3A%2F%2Fdata.humdata.org%2Fdataset%2Fstructures_de_sante_guinee_vf%2Fresource%2F3d5e7ab6-5164-457d-bcc8-3cc1aa9b01aa__%3B!!OT8QuQ!saYZgZ8VEHQ5Mbs14z5iaNgMOgnQfNGE8En5ZlBDHd4MS10-9fgdbIpCjb03EHXC8JaKffVOwUUReDRE4wCmTevK58sLAA%24&data=05%7C01%7Cmverykiou%40gavi.org%7C8bf6d872566c42da509c08db6b2461ad%7C1de6d9f30daf4df6b9d65959f16f6118%7C0%7C0%7C638221576224359928%7CUnknown%7CTWFpbGZsb3d8eyJWIjoiMC4wLjAwMDAiLCJQIjoiV2luMzIiLCJBTiI6Ik1haWwiLCJXVCI6Mn0%3D%7C3000%7C%7C%7C&sdata=7xVQzwhgf8g9Dv%2BwV7bdAHMtA3mvtGNgx23WlecO5NE%3D&reserved=0) [↑](#footnote-ref-5)
5. Ikilezi G, Micah AE, Bachmeier SD, Cogswell IE, Maddison ER, Stutzman HN, Tsakalos G, Brenzel L, Dieleman JL. Estimating total spending by source of funding on routine and supplementary immunisation activities in low-income and middle-income countries, 2000–17: a financial modelling study. The Lancet. 2021 Nov 20;398(10314):1875-93. [↑](#footnote-ref-6)
